# Association Between Argatroban and Outcomes of Branch Atheromatous Disease: A Propensity‐Matched Analysis From MRI‐Based Study

**DOI:** 10.1111/cns.70467

**Published:** 2025-06-17

**Authors:** Shengde Li, Haizhou Hu, Yaping Zhou, Ping Zhang, Guofang Chen, Hongying Bai, Bin Liu, Lixin Zhou, Yicheng Zhu, Bin Peng, Jun Ni, BAD‐study investigators

**Affiliations:** ^1^ Department of Neurology State Key Laboratory of Complex Severe and Rare Diseases, Peking Union Medical College Hospital, Chinese Academy of Medical Science and Peking Union Medical College Beijing China; ^2^ Department of Neurology The First Affiliated Hospital of Xinxiang Medical University Weihui Henan China; ^3^ Department of Neurology Xuzhou Central Hospital Xuzhou Jiangsu China; ^4^ Department of Neurology The Second Affiliated Hospital of Zhengzhou University Zhengzhou Henan China; ^5^ Department of Neurology Affiliated Hospital of North China University of Science and Technology Tangshan Hebei China

**Keywords:** argatroban, arterial occlusive diseases, atherosclerosis, prognosis, stroke

## Abstract

**Background and Purpose:**

Argatroban is widely used for patients with acute branch atheromatous disease (BAD)‐related stroke, but its efficacy remains unclear. This study aims to evaluate its clinical outcomes in this patient population.

**Methods:**

A prospective, MRI‐based cohort (BAD‐study) was conducted across 20 hospitals in China from June 2021 to June 2023, enrolling patients aged 18–80 years with BAD‐related stroke within 72 h of onset. Patients were divided into two groups: Argatroban and non‐argatroban. The primary outcome was an excellent outcome, defined as a modified Rankin Scale (mRS) score of 0–1 at 90 days. Secondary outcomes included good outcome (mRS 0–2), mRS score, Barthel Index score at 90 days, and NIHSS score at 7 days. Logistic regression analyses were performed to assess the association between argatroban and outcomes after propensity score matching (PSM).

**Results:**

A total of 467 patients were included, with a median age of 60 years and a median NIHSS score of 4 at admission. Eighty‐six patients (18.4%) were in the argatroban group, and 381 patients (81.6%) were in the non‐argatroban group. After PSM, excellent outcomes occurred in 60.0% of the argatroban group and 64.2% of the non‐argatroban group (odds ratio [OR] = 0.84, 95% CI: 0.47–1.49, *p* = 0.542). Argatroban was not significantly associated with secondary outcomes and remained ineffective in sensitivity analyses.

**Conclusion:**

Argatroban was not associated with excellent outcome at 90 days in patients with acute BAD‐related stroke. Our study suggests that the risks and benefits of argatroban need reevaluation in patients with BAD‐related stroke.

## Graphical abstract

In patients with branch atheromatous disease‐related stroke, argatroban was not associated with excellent outcome (mRS 0–1) at 90 days, though it was widely used. The risks and benefits of argatroban need reevaluation in this patient population.

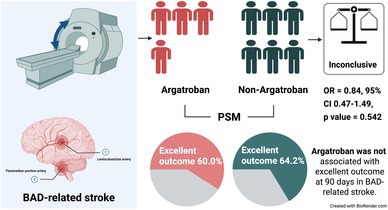

## 1 Introduction

Branch atheromatous disease (BAD) accounts for 9.1%–18.3% of cases of acute ischemic stroke (AIS) in Asia [1, 2], with a high risk of early neurological deterioration (END) and disability [2, 3, 4]. The disability rate in BAD‐related stroke can reach up to 61%, significantly higher than that observed in lacunar infarction [2]. Although BAD is not characterized by severe large artery stenosis, the rate of END in BAD‐related stroke is similar to that seen in large‐artery atherosclerosis (LAA) [5, 6]. Current guidelines recommend early intravenous thrombolysis to reduce disability in ischemic stroke [7]; however, this treatment does not prevent END in BAD‐related stroke nor improve functional outcomes at 90 days [8]. Moreover, patients with BAD‐related stroke are ineligible for endovascular therapy, as BAD results from proximal stenosis or occlusion of a penetrating artery, not from severe stenosis of the parent artery [3, 9]. As a result, several acute thrombotic agents, including dual antiplatelet therapy, tirofiban, cilostazol, and argatroban, have been explored for improving outcomes in BAD‐related stroke [10, 11, 12, 13]. However, the efficacy of these therapies remains inconsistent, and most evaluations have been limited to small sample sizes or retrospective designs [10, 12, 13]. The need for MRI during the acute phase to diagnose BAD‐related stroke further complicates randomized controlled trials (RCTs) in this context. In the absence of an optimal acute‐phase treatment, clinicians often resort to various pharmacological interventions with limited evidence [14].

Argatroban, a direct thrombin inhibitor, is thought to prevent thrombus propagation, potentially benefiting patients with ischemic stroke [15]. While argatroban significantly increased favorable outcomes (mRS 0–3) from 73.3% to 80.5% in AIS patients with END, it did not reduce disability in patients treated with intravenous thrombolysis and was even associated with increased mortality [16, 17, 18]. A systematic review also failed to support the adjunctive use of argatroban following thrombolysis [19]. Recently, a small‐sample randomized trial suggested that argatroban plus dual antiplatelet therapy could benefit patients with mild BAD‐related stroke [13]. In clinical practice, 51.2% of patients with BAD‐related stroke and 18.5% of those with lacunar infarction received argatroban [20]. Neurologists may hypothesize that argatroban improves the outcomes of BAD‐related stroke, but robust evidence supporting its use is lacking.

Therefore, we conducted a multicenter, prospective, observational study to evaluate the efficacy of argatroban in patients with BAD‐related stroke in a real‐world setting.

## 2 Method

### 2.1 Study Design and Population

Patients were recruited from the BAD study, a multicenter, observational, prospective cohort study conducted across 20 hospitals in China from June 2021 to June 2023. Details of the study design and the BAD study have been published elsewhere [21]. Patients aged 18–80 years with BAD‐related stroke within 72 h of symptom onset were included, provided they met the radiological criteria for BAD: (1) Lesion on diffusion‐weighted imaging (DWI): single (isolated) deep (subcortical) infarct; (2) Culprit vessels were lenticulostriate artery (LSA) or paramedian pontine artery (PPA), and the infarct lesion on diffusion‐weighted imaging conformed to one of the following characteristics (LSA/PPA): LSA: comma‐like infarct lesions with fan‐shaped extension from bottom to top in the coronal plane or ≥ 3 layers (layer thickness, 5–7 mm) on axial DWI, or PPA: infarct lesion extended from the deep pons to the ventral pons on the axial DWI; and (3) no ≥ 50% stenosis on the parent artery of the criminal vessel. The main exclusion criteria were: (1) ≥ 50% stenosis of extracranial vessels with ipsilateral serial relationship; (2) cardiogenic embolism. The details of enrollment criteria were published elsewhere [21]. Diagnostic criteria of BAD were consistent across centers in China. The BAD study was approved by the ethics committee of Peking Union Medical College Hospital (ZS‐2982B), and written informed consent was obtained from all participants. The BAD study has been registered at https://clinicaltrials.gov/, with identifier NCT 04973774.

### 2.2 Clinical Assessment and Outcomes

Demographic, clinical, radiologic, laboratory, and therapeutic data were prospectively collected at baseline and follow‐up. The National Institutes of Health Stroke Scale (NIHSS), modified Rankin Scale (mRS), and Barthel Index scores were also recorded [21]. Obesity was defined as a body mass index ≥ 28 kg/m^2^ [22]. The primary outcome was excellent outcome, defined as an mRS score of 0–1 at 90 days. Secondary outcomes included good outcome (mRS: 0–2), mRS score, Barthel Index score, vascular events (new‐onset myocardial infarction, ischemic stroke, or intracranial hemorrhage), and bleeding events at 90 days. NIHSS scores at 7 days were also recorded.

**FIGURE 1:**
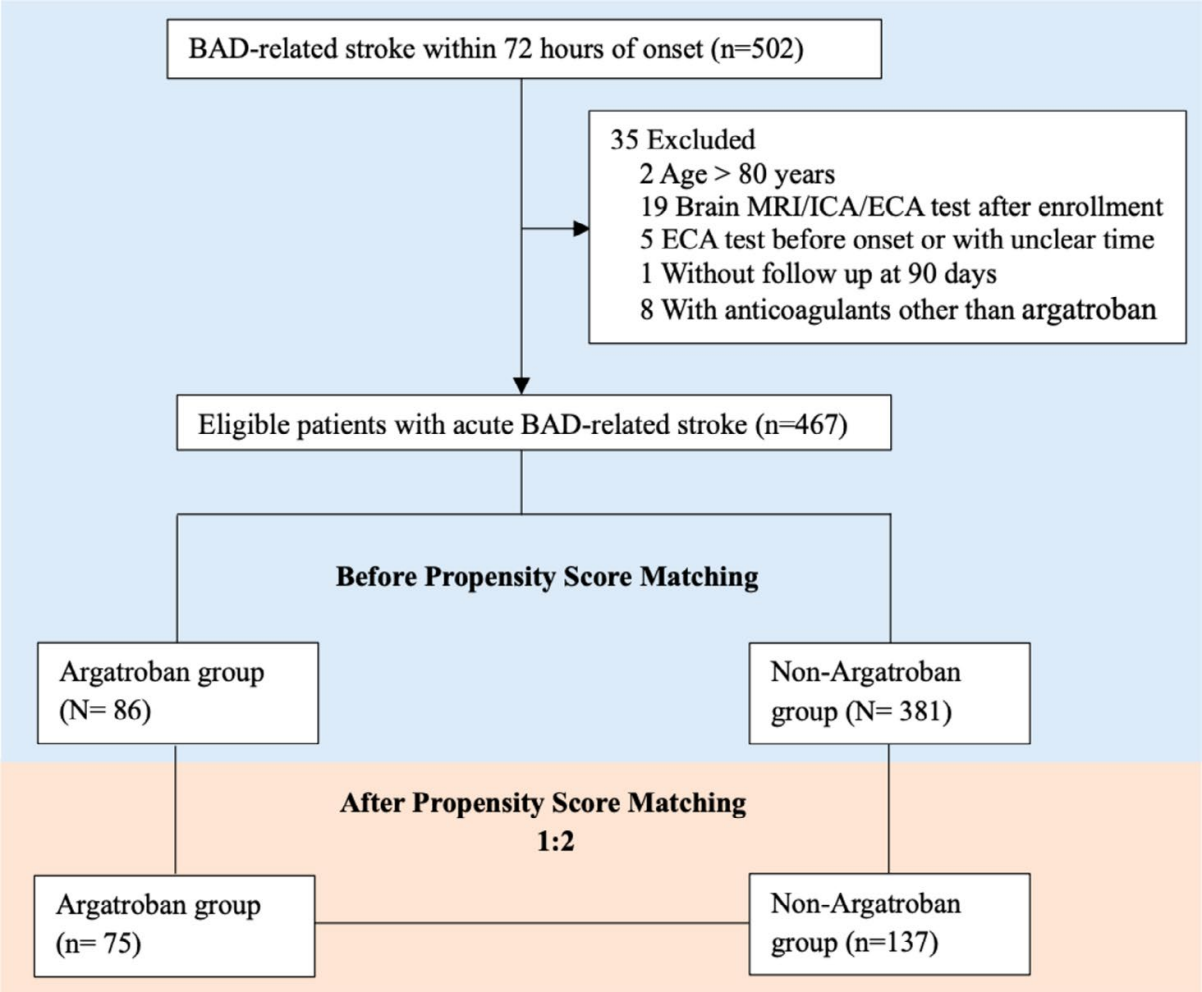
Patients’ flow of our study.

From symptom onset to 7 days after enrollment, patients in the BAD study received either argatroban or non‐argatroban treatment, as decided by the patient or their guardian following a thorough discussion with a neurologist (Figure 1). The argatroban group received continuous intravenous argatroban at 60 mg per day for the first 2 days and 20 mg per day for the next 5 days.

Early neurological deterioration (END) was defined as follows: (1) a ≥ 4 point increase in NIHSS score, or a ≥ 1 point increase in NIHSS motor score for ischemic stroke; (2) ≥ 3 stereotyped attacks after admission or progression to persistent status for internal capsule/pontine warning syndrome for transient ischemic attack; (3) END was evaluated from symptom onset to 7 days after enrollment [23, 24].

### 2.3 Statistical Analysis

Categorical variables are presented as frequencies and percentages, and comparisons between the argatroban and non‐argatroban groups were made using χ^2^ tests. The Shapiro–Wilk test was used to assess the normality of variables. Continuous variables with non‐normal distributions were compared using Mann–Whitney tests and are presented as medians with interquartile ranges (IQR). A propensity score matching (PSM) analysis using logistic regression was performed to generate balanced cohorts. Baseline variables with an absolute standardized mean difference (SMD) greater than 0.1 were included in the matching process. A 1:2 greedy nearest neighbor matching mode with a caliper width of 0.1 was applied. Variables included in the PSM were: age, sex, criminal artery, extracranial artery stenosis, NIHSS score at admission, mRS score at enrollment, intravenous tirofiban, dual antiplatelet therapy, smoking, and END. The balance of covariates was assessed using standardized mean differences. For variables with SMD ≥ 0.1 after PSM, P‐values were calculated using χ^2^ or Mann–Whitney tests. Logistic regression models were used to compare functional outcomes, including NIHSS scores, mRS scores, and Barthel Index scores at 90 days, between matched cohorts. NIHSS scores at 7 days were also assessed using logistic regression. Additionally, Cox proportional hazards regression was used to compare the risk of vascular and bleeding events between the matched cohorts.

Missing mRS scores at enrollment were imputed using the median mRS score, stratified by NIHSS score at admission (Table S1). Obesity was excluded from the PSM analysis due to missing data. To reduce bias, a multivariable logistic regression analysis was conducted, excluding missing data for mRS and BMI. In addition, to assess the preventive effect of argatroban, a sensitivity analysis restricted to patients who received argatroban before the occurrence of END was performed. A subgroup analysis was performed based on the occurrence of END and the use of intravenous thrombolysis to assess the efficacy of argatroban in BAD‐related stroke.

All statistical tests were two‐sided, with a P‐value of < 0.05 considered statistically significant. Standardized mean difference and PSM process were conducted using R version 4.1.3 (DescTools and MatchIt). The other analyses were performed using SAS version 9.4 (SAS Institute, Cary, NC).

## 3 Results

The initial cohort included 502 patients with acute BAD‐related stroke. Of these, 26 were excluded due to violations of enrollment criteria, and 9 were excluded due to loss to follow‐up or use of anticoagulants other than argatroban. Therefore, 467 patients were included in the final analysis (Figure 1). The median age was 60 years (IQR: 53–68), and 29.3% were female. The median NIHSS score at admission was 4 (IQR: 2–6). In total, 86 (18.4%) patients were in the argatroban group, and 381 (81.6%) were in the non‐argatroban group.

Before propensity score matching (PSM), the argatroban group had higher NIHSS scores at admission, NIHSS scores at enrollment, and mRS scores at enrollment. Additionally, the argatroban group had a higher rate of extracranial artery stenosis and END, and a lower rate of dual antiplatelet therapy and obesity compared to the non‐argatroban group (Table 1).

**TABLE 1:** Basic demographics and clinical data of patients before and after PSM.

	Before propensity score match				After propensity score match		
	All (*n* = 467)	Non‐argatroban (*n* = 381)	Argatroban (*n* = 86)	SMD	Non‐argatroban (*n* = 137)	Argatroban (*n* = 75)	SMD
Age	60 (53–68)	60 (53–68)	61 (54–67)	0.053	62 (54–69)	61 (54–67)	0.039
Sex, female	137 (29.3)	114 (29.9)	23 (26.7)	0.070	34 (24.8)	22 (29.3)	0.102
Type of symptom				0.084			0.026
Motor symptoms	367 (78.6)	297 (78.0)	70 (81.4)		110 (80.3)	61 (81.3)	
Others	100 (21.4)	84 (22.0)	16 (18.6)		27 (19.7)	14 (18.7)	
NIHSS at admission	4 (2–6)	3 (2–6)	4 (3–7)	0.291	4 (2–7)	4 (3–7)	0.076
NIHSS score at enrollment	3 (2–6)	3 (2–6)	5 (3–7)	0.468	4 (2–7)	5 (3–7)	0.095
mRS score at enrollment	2 (1–3)	2 (1–3)	3 (2–4)	0.633	3 (2–4)	3 (2–4)	0.070
Barthel index at enrollment	80 (60–100)	85 (65–100)	70 (50–85)	0.502	73 (55–90)	70 (50–85)	0.038
SBP at admission	151 (138–165)	151 (138–165)	152 (140–165)	0.087	149 (137–163)	152 (139–165)	0.077
DBP at admission	90 (80–100)	90 (80–100)	90 (81–102)	0.159	90 (79–100)	90 (80–101)	0.192
Hypertension	253 (54.2)	207 (54.3)	46 (53.5)	0.017	76 (55.5)	42 (56.0)	0.011
Diabetes	113 (24.2)	93 (24.4)	20 (23.3)	0.027	37 (27.0)	16 (21.3)	0.131
LDL‐C	2.87 (2.33–3.41)	2.89 (2.33–3.48)	2.76 (2.36–3.30)	0.113	2.88 (2.30–3.44)	2.80 (2.36–3.30)	0.057
History of stroke	83 (17.8)	68 (17.9)	15 (17.4)	0.011	22 (16.1)	13 (17.3)	0.034
Coronary heart disease	31 (6.6)	28 (7.4)	3 (3.5)	0.155	11 (8.0)	3 (4.0)	0.162
Obesity*	68 (14.6)	63 (16.6)	5 (5.8)	0.307	27 (20.0)	4 (5.3)	0.419
Smoking, ever	190 (40.7)	162 (42.5)	28 (32.6)	0.203	51 (37.2)	24 (32.0)	0.109
Regular drinking	117 (25.1)	95 (24.9)	22 (25.6)	0.015	39 (28.5)	19 (25.3)	0.070
Criminal artery				0.022			0.021
LSA	281 (60.2)	230 (60.4)	51 (59.3)		80 (58.4)	43 (57.3)	
PPA	186 (39.8)	151 (39.6)	35 (40.7)		57 (41.6)	32 (42.7)	
Stenosis of ICA	169 (36.2)	143 (37.5)	26 (30.2)	0.152	47 (34.3)	21 (28.0)	0.135
Stenosis of ECA	118 (25.3)	89 (23.4)	29 (33.7)	0.239	41 (29.9)	25 (33.3)	0.073
Hours from onset to admission	15 (6–24)	16 (6–25)	13 (6–24)	0.206	17 (6–25)	13 (6–24)	0.208
Hours from onset to enrollment	49 (40–64)	49 (40–65)	48 (37–59)	0.112	49 (40–67)	48 (36–59)	0.168
Intravenous thrombolysis	74 (15.9)	59 (15.5)	15 (17.4)	0.053	23 (16.8)	10 (13.3)	0.095
Dual antiplatelet therapy	302 (64.7)	257 (67.5)	45 (52.3)	0.318	82 (59.9)	41 (54.7)	0.105
Use of statins	438 (93.8)	357 (93.7)	81 (94.2)	0.020	127 (92.7)	71 (94.7)	0.079
Use of intravenous tirofiban	75 (16.1)	61 (16.0)	14 (16.3)	0.007	27 (19.7)	13 (17.3)	0.060
END	69 (14.8)	40 (10.5)	29 (33.7)	0.675	32 (23.4)	18 (24.0)	0.015

*Note:* Continuous variables with non‐normal distribution are shown as median (interquartile range); other values are presented as *n* (%).

Abbreviations: DBP, diastolic blood pressure; ECA, extracranial carotid artery; END, early neurological deterioration; ICA, intracranial artery; LDL‐C, low‐density lipoprotein cholesterol; LSA, lenticulostriate artery; mRS, modified Rankin Scale; NIHSS, National Institutes of Health Stroke Scale; PPA, paramedian pontine artery; PSM, propensity score matching; SBP, systolic blood pressure; SMD, standardized mean difference. *Missing data: 2 cases without body mass index data.

After PSM, 75 patients were in the argatroban group and 137 in the non‐argatroban group (Table 1, Figure 1). Standardized mean differences (SMDs) for variables after PSM are shown in Table 1. For variables with an SMD ≥ 0.1, *p*‐values were > 0.05, except for obesity (*p* = 0.04) (Table S2). After PSM, the rates of excellent outcomes were 60.0% in the argatroban group and 64.2% in the non‐argatroban group, with no statistically significant difference (*p* = 0.542). The logistic regression model showed that argatroban was not associated with excellent outcomes (OR = 0.84, 95% CI: 0.47–1.49, *p* = 0.542) (Table 2).

**TABLE 2:** Associations between argatroban and outcomes after PSM.

Outcome	Non‐argatroban (*n* = 137)	Argatroban (*n* = 75)	*p*	OR/HR (95% CI)	*p*
Excellent outcome	88 (64.2)	45 (60.0)	0.542	0.84 (0.47–1.49)	0.542
Good outcome	115 (83.9)	61 (81.3)	0.629	0.83 (0.40–1.74)	0.629
mRS score at day 90	1 (0–2)	1 (0–2)	0.356	0.79 (0.48–1.31)	0.356
Barthel index score at day 90	100 (90–100)	95 (85–100)	0.159	1.45 (0.86–2.44)	0.161
NIHSS at day 7	4 (2–6)	4 (2–7)	0.681	0.90 (0.55–1.48)	0.687
Vascular events	1 (0.7)	1 (1.3)	1.0000	NA*	0.998
Bleeding	0 (0)	0 (0)	NA	NA*	NA

*Note:* mRS score at day 90: ordinal logistic regression for a shift in the direction of worse outcome on the modified Rankin scale by very 1 point. Barthel index score at day 90: ordinal logistic regression for a shift in the direction of better outcome on the Barthel index by very 5 points. NIHSS at day 7: ordinal logistic regression for a shift in the direction of worse outcome on NIHSS by very 1 point.

Abbreviations: HR, hazard ratio; mRS, modified Rankin Scale; NIHSS, National Institutes of Health Stroke Scale; OR, odds ratio.

*Hazard ratio; others for odds ratio. NA, for unavailable data.

For secondary outcomes, there was no significant association between argatroban and good outcomes, mRS scores, or Barthel Index scores at 90 days. NIHSS scores at 7 days were similar between the groups (OR = 0.90, 95% CI: 0.55–1.48, *p* = 0.687). The risk of vascular (0.7% vs. 1.3%) and bleeding (0% vs. 0%) events was low and comparable between the groups (Table 2).

### 3.1 Sensitivity Analysis

We excluded 12 patients with missing mRS data at enrollment, and a multivariable logistic regression analysis of the remaining 455 patients showed that argatroban was not associated with excellent outcomes at 90 days (OR = 0.79, 95% CI: 0.42–1.46, *p* = 0.448). After excluding 14 patients with missing data for both mRS and obesity, logistic regression, adjusted for age, sex, NIHSS score, mRS score, END, intravenous thrombolysis, dual antiplatelet therapy, smoking, extracranial carotid artery stenosis, criminal artery, and obesity, confirmed that argatroban did not predict excellent outcomes in BAD‐related stroke (OR = 0.79, 95% CI: 0.42–1.48, *p* = 0.461).

After excluding 18 patients treated with argatroban after END occurrence, argatroban was not associated with excellent outcome at 90 days (OR = 0.75, 95% CI: 0.39–1.45, *p* = 0.390) (Table 3).

No significant association was found between argatroban and excellent outcomes in the following four subgroups: with END, without END, with intravenous thrombolysis, and without intravenous thrombolysis (Table 3).

**TABLE 3:** Sensitivity analysis for excellent outcome in patients with BAD‐related stroke.

Models	Non‐argatroban *n*/*N* (%)	Argatroban *n*/*N* (%)	*p*	OR (95% CI)	*p*
Model 1	278/369 (75.3)	47/86 (54.7)	< 0.001	0.79 (0.42–1.46)	0.448
Model 2	277/367 (75.5)	47/86 (54.7)	< 0.001	0.79 (0.42–1.48)	0.461
Model 3	258/328 (78.7)	38/57 (66.7)	0.048	0.95 (0.44–2.05)	0.899
Model 4	19/39 (48.7)	9/29 (31.0)	0.143	0.52 (0.15–1.90)	0.325
Model 5	230/311 (74.0)	41/71 (57.8)	0.007	1.05 (0.52–2.15)	0.886
Model 6	47/56 (83.9)	6/15 (40.0)	0.001	0.51 (0.06–4.54)	0.547
Model 7	289/381 (75.9)	42/68 (61.8)	0.015	0.75 (0.39–1.45)	0.390

*Note:* Model 1 was adjusted for age, sex, NIHSS score at admission, mRS score at enrollment, END, intravenous thrombolysis, dual antiplatelet therapy, smoking, stenosis of extracranial carotid artery, and criminal artery; 455 patients were included. Model 2 was adjusted for age, sex, NIHSS score at admission, mRS score at enrollment, END, intravenous thrombolysis, dual antiplatelet therapy, smoking, stenosis of extracranial carotid artery, criminal artery and obesity; 453 patients were included. Model 3 included patients without END (*n* = 385), and was adjusted for age, sex, NIHSS score at admission, mRS score at enrollment, intravenous thrombolysis, dual antiplatelet therapy, smoking, stenosis of extracranial carotid artery, criminal artery and obesity. Model 4 included patients with END (*n* = 68), and was adjusted for age, sex, NIHSS score at admission, mRS score at enrollment, intravenous thrombolysis, dual antiplatelet therapy, smoking, stenosis of extracranial carotid artery, criminal artery, and obesity. Model 5 patients without intravenous thrombolysis (*n* = 382), and was adjusted for age, sex, NIHSS score at admission, mRS score at enrollment, END, dual antiplatelet therapy, smoking, stenosis of extracranial carotid artery, criminal artery, and obesity. Model 6 included patients with intravenous thrombolysis (*n* = 71), and was adjusted for age, NIHSS score at admission, mRS score at enrollment, END, and obesity. Model 7 was adjusted for age, sex, NIHSS score at admission, mRS score at enrollment, END, intravenous thrombolysis, dual antiplatelet therapy, smoking, stenosis of extracranial carotid artery, and criminal artery. Model 7 excluded patients who received argatroban after the occurrence of END (*n* = 18), and 449 patients were analyzed.

Abbreviation: OR, odds ratio.

## 4 Discussion

In this multicenter, observational, prospective cohort study, we found that the initiation of intravenous argatroban within 7 days of enrollment in patients with acute BAD‐related stroke was not associated with an excellent outcome at 90 days. This finding aligns with previous studies, which also showed no improvement in outcomes for AIS patients treated with argatroban, whether combined with alteplase or endovascular therapy [17, 18, 25]. Our study further confirms the lack of benefit of argatroban in BAD‐related stroke treated with intravenous thrombolysis.

Interestingly, a randomized trial by Zhang et al. in AIS patients with END found that argatroban increased the rate of favorable outcomes (mRS 0–3 at 90 days) (risk ratio = 1.10, 95% CI: 1.01–1.20), although no significant difference was observed in the mRS 0–2 category [16]. These findings might be explained by differences in baseline characteristics, as nearly half of Zhang's patients had large‐artery atherosclerosis (LAA), with higher NIHSS scores at baseline compared to our cohort (8 vs. 4). Other studies suggest that argatroban may be more effective in moderate to severe AIS, especially those resulting from LAA, rather than in BAD‐related stroke [17, 18]. For example, Yan et al. reported that argatroban improved prognosis in AIS patients with LAA and NIHSS scores ≥ 5 [26].

BAD‐related stroke presents a unique challenge due to its high risk of END, a predictor of poor long‐term outcomes [3, 4]. Several studies have suggested that dual antiplatelet therapy combined with argatroban may help prevent END in BAD‐related stroke or posterior circulation AIS [10, 27, 28]. However, conflicting results have emerged, including a post hoc analysis of the ARAIS trial indicating that argatroban plus alteplase could reduce END in patients with NIHSS scores ≥ 10 [29]. But a recent randomized trial of BAD‐related stroke found that argatroban plus dual antiplatelet therapy reduced END occurrence and improved clinical outcomes in patients with a median NIHSS score of 2 [13]. These inconsistencies underscore the need for further large‐sample trials to definitively determine the role of argatroban in this patient population.

There were differences between our study and that by Xu et al., which may explain the divergent findings. Our cohort had a higher baseline NIHSS score compared to theirs (4 vs. 2), and our study featured a lower rate of dual antiplatelet therapy. Our intensity of antithrombotic therapy was lower [13]. Moreover, the efficacy of argatroban in BAD‐related patients with a NIHSS score ≥ 6 remains undetermined, who were at higher risk of disability [18, 30]. These factors could potentially influence the outcome and warrant further investigation.

Most previous large‐sample randomized trials of argatroban focused on broader populations of ischemic stroke, with baseline NIHSS scores ranging from 8 to 12 and a main etiology of small‐vessel occlusion, large‐artery atherosclerosis, or undetermined [16, 17, 18]. Argatroban was initiated within 4.5 or 3 h of stroke onset in patients receiving intravenous thrombolysis, or 48 h in patients suffering END [16, 17, 18]. In contrast, our cohort included patients with milder strokes due to BAD etiology, and argatroban was initiated later, which may explain the lack of benefit observed in our study.

While we observed a lower proportion of obesity in the argatroban group, we adjusted for this in our analysis and found that obesity did not significantly influence the efficacy of argatroban. Previous studies have similarly shown that BMI or obesity does not significantly affect functional outcomes in AIS [31, 32]. Thus, the impact of obesity on our findings was likely minimal.

Our study has several limitations. First, the therapy assignment was nonrandomized. Although PSM was used to reduce confounding, residual imbalances may remain. Factors such as the timing of argatroban administration, clinical decision‐making complexity, and potential interactions with other therapies may have influenced outcomes. These real‐world dynamics, not fully captured by matching, could partly explain the observed differences in functional outcomes. Second, although subgroup analyses by END and intravenous thrombolysis showed no significant benefit, the potential synergy between argatroban, antiplatelet agents, and alteplase warrants further investigation [10, 19]. Furthermore, as argatroban was prescribed at the neurologist's discretion, our study compared treatment strategies that included or excluded it rather than the efficacy of argatroban as a standalone therapy. Finally, as argatroban was administered both before and after END, variability in timing—especially delayed initiation—may have attenuated its effectiveness and further complicated outcome interpretation.

In conclusion, intravenous argatroban was not associated with an excellent outcome at 90 days in patients with acute BAD‐related stroke. Neurologists should carefully reconsider the risks and benefits of using argatroban in patients with BAD‐related stroke, particularly in light of the lack of clear evidence supporting its efficacy in this patient population.

## Author Contributions

S.L. designed and wrote the manuscript. H.H, Yaping Z., H.B., B.L., G.C., L.Z., and P.Z. revised the manuscript. Yicheng Z., B.P., and J.N. gave constructive advice and participated in the proofreading of this paper. All authors contributed to the article and approved the submitted version.

## Conflicts of Interest

The authors declare no conflicts of interest.

## Supporting information


**Table S1.** Input for missing data of mRS at enrollment.
**Table S2.** *P* values for variables with standardized mean difference ≥ 0.1 after propensity score matching.

## Data Availability

The data that support the findings of this study are available from the corresponding author upon reasonable request.
