# Supplementary material for: Association Between Argatroban and Outcomes of Branch Atheromatous Disease: A Propensity‐Matched Analysis From MRI‐Based Study
Source: CNS Neurosci Ther. 2025 Jun 17;31(6):e70467. doi: 10.1111/cns.70467 (PMC12174617; doi:10.1111/cns.70467)
Supplement: Supplementary file 1 — Table S1. Input for missing data of mRS at enrollment. Table S2. P values for variables with standardized mean difference ≥ 0.1 after propensity score matching. [file CNS-31-e70467-s001.docx]

**Supplementary material**

**Table S1. Imput for missing data of mRS at enrollment**

| NIHSS at admission from those without missing data of mRS | Median mRS score | Imputed mRS | Number of patients |
| --- | --- | --- | --- |
| 0 | 1 (0-1) | 1 | 1 |
| 2 | 1 (1-2) | 1 | 4 |
| 3 | 2 (1-3) | 2 | 3 |
| 5 | 2 (2-3) | 2 | 1 |
| 6 | 3 (2-4) | 3 | 2 |
| 8 | 4 (3-4) | 4 | 1 |

mRS, modified Rankin Scale; NIHSS, National Institutes of Health Stroke Scale.

**Table S2. P values for variables with standardized mean difference ≥ 0.1 after propensity score matching**

| **Variables** | **P value** |
| --- | --- |
| Sex | 0.476 |
| DBP at admission | 0.227 |
| Diabetes | 0.362 |
| Coronary heart disease | 0.387 |
| Obesity* | 0.004 |
| Smoking, ever | 0.447 |
| Stenosis of ICA | 0.347 |
| Hours from onset to admission | 0.148 |
| Hours from onset to enrollment | 0.686 |
| Dual antiplatelet therapy | 0.464 |

For obesity and coronary heart disease, Fisher's exact tests were used.

*Missing data: 2.
